# The habitual, spatial and temporal conditions of everyday youth intergroup contact in an ethnically diverse city

**DOI:** 10.1111/bjso.70075

**Published:** 2026-04-05

**Authors:** Sumedh Rao, Pier‐Luc Dupont, David Manley, Laura K. Taylor, Shelley McKeown

**Affiliations:** ^1^ The Open University Milton Keynes UK; ^2^ Swansea University Swansea UK; ^3^ University of Bristol Bristol UK; ^4^ University College Dublin Dublin Ireland; ^5^ University of Oxford Oxford UK

**Keywords:** ethnic diversity, intergroup contact, prejudice reduction, urban space, youth interactions

## Abstract

There is substantial evidence that positive intergroup contact can reduce prejudice. Most everyday interactions, however, are not deliberately structured to be positive, and individuals do not always engage in intergroup contact even when there is opportunity. The present research adopts a qualitative approach to understand how youth negotiate everyday contact with outgroup friends and acquaintances in the ethnically diverse city of Bradford, England. We explore how youth intergroup interactions manifest in everyday life, how urban spaces facilitate or inhibit them, and the psychological processes involved. A total of 33 youth aged 16–18 (16 Asian, 14 White, 1 Black, 1 Arab, 1 mixed race) took part in a photography project and focus group sessions, and nine of those youth (4 Asian, 3 White, 1 Black, 1 Arab) took part in follow‐up walking interviews. Data were analysed using reflexive thematic analysis. Findings demonstrated the habitual nature of everyday intergroup contact and the complex negotiations youth engage in to socialise with outgroup friends. They also highlight how space perceptions influence the maintenance of cross‐ethnic friendships and are shaped by past experiences and memories. Our research has implications for understanding everyday unstructured interactions and the spatial and temporal factors that influence youth intergroup contact.

Intergroup contact, the process of bringing groups together in a positive and meaningful way (Allport, 1954), is often hailed as one of the most effective ways to reduce prejudice. While there is substantial empirical support for the basic premise of intergroup contact theory (Pettigrew & Tropp, 2006), the majority of related research has explored structured and somewhat idealised forms of contact through fixed measures of contact quantity and quality (Dixon et al., 2005). By contrast, a significant proportion of contact experiences is unstructured and banal, especially in everyday urban spaces. We define these spaces as physical and social environments, such as parks, shopping centres, cafés and leisure centres, where routine activities unfold and intergroup encounters can occur (cf. the ‘social infrastructures’ studied by Wessendorf & Gembus, 2024, p. 2826). Although several scholars have examined everyday intergroup contact *in situ* (e.g., Bettencourt et al., 2019; Dixon et al., 2025; Paajanen et al., 2022; Wessendorf & Gembus, 2024), research has rarely focused on the psychological processes that underpin interactions within diverse friendship groups in everyday public spaces. Moreover, while prior studies have documented where and when contact occurs, they have not fully theorised how spatial and temporal dynamics shape these interactions. Our study addresses these gaps in two ways. First, we explore how youth intergroup interactions manifest in everyday spaces and how they become habitual. Second, we introduce a spatial and temporal perspective on everyday contact among diverse friends. To achieve this, we adopt a qualitative approach and focus specifically on youth everyday intergroup contact in the city of Bradford, England.

## Everyday intergroup contact

Intergroup contact theory has long emphasised the importance of interactions, ideally under the conditions of equal status, common goals, cooperation and social/institutional support, in reducing prejudice and fostering positive intergroup relations (Allport, 1954; Pettigrew & Tropp, 2006). Despite substantial empirical evidence demonstrating the effectiveness of structured intergroup contact, researchers have highlighted a disconnect between the optimal interactions usually studied in the laboratory or in localised interventions and the nature of most contact taking place in everyday life (Dixon et al., 2005). That is, much of the intergroup contact people experience is unregulated, mundane and superficial, which makes it ill‐suited for the intimate self‐disclosure that has been shown to catalyse attitudinal change (Pettigrew, 1998). The social psychological perspective on the effectiveness of everyday contact in reducing prejudice is therefore a sceptical one: to paraphrase Dixon et al. (2005), exploring contact ‘*in situ*’ offers a ‘reality check’ on the rosier picture painted by research on structured contact. This scepticism has been less pronounced in other disciplines such as urban and cultural geography, anthropology and sociology. In line with Dixon's (2016) renewed call to study contact ‘in the wild’, researchers of ‘conviviality’ (Nowicka & Vertovec, 2014), intercultural ‘encounters’ (Valentine, 2008), ‘commonplace diversity’ (Wessendorf, 2014), ‘multiculture’ (Neal et al., 2018) and ‘everyday multiculturalism’ (Wise & Velayutham, 2009) have conducted ethnographies and semi‐structured interviews in a variety of public and semi‐public spaces such as parks, playgrounds, shops, markets, restaurants, streets, buses, post offices, sports centres, nightclubs, libraries, community centres and churches. Focusing mostly on superficial intergroup interactions between strangers or acquaintances, these studies have offered rich analyses of spontaneous and unremarkable forms of cooperation, from smiling and small talk to holding a door open, responding to a query and helping with simple tasks. Yet this literature's emphasis on the capacity of (semi‐)public spaces to generate new intergroup connections has led to a relative neglect of their role in sustaining already existing and usually much more meaningful intergroup friendships. It is this habitual nature of contact, the recurring interactions that unfold in familiar spaces with familiar people, that we focus on in the present research.

## Habitual, spatial and temporal aspects of everyday contact

Understanding habitual patterns of behaviour, including how people navigate their daily life, with whom and where is crucial to understanding intergroup contact. Routines are learned and become automatic through repeated practice over time in specific contexts (Verplanken & Aarts, 1999). They can either facilitate or inhibit intergroup contact by shaping the choices individuals make about where to go, who to sit with, or how to navigate shared urban spaces (Dixon et al., 2005; Reimer, 2018). These mundane decisions are not necessarily deliberate but rather stem from comfort, familiarity and prior relational histories with people and in spaces. These routine habits can sometimes reinforce group‐based homophily and reduce intergroup contact, particularly in segregated societies (McPherson et al., 2001).

While habits can be difficult to change, routine use of space and, in turn, the people we are exposed to can be disrupted through events and life transitions. The move from school to college or from university to work, for example, can open up new social interaction opportunities through changing the context in which time is spent (Meleady et al., 2025; Paolini et al., 2026). However, even in diverse settings, preferences for same‐ethnic friendships and habitual ties to pre‐existing friendship networks may persist, limiting intergroup contact engagement (Mouw & Entwisle, 2006).

As noted above, people do not only live out everyday contact habits in highly regulated sites such as schools and workplaces but also in the wider built environment or social infrastructure. In urban studies, such spaces are known as *servicescapes*, representing the physical and social environments in which services (both public and private) are delivered and experienced (Bitner, 1992). Servicescapes are routinely accessed spaces that can offer opportunities for fleeting encounters with diverse others as well as attractive and safe spaces where existing cross‐group friendships can be maintained. For example, individuals might meet diverse others whilst paying the bill in a restaurant or spend time with outgroup friends in a city park, therefore gaining opportunities for varied types and levels of intercultural dialogue (Amin, 2002).

People's use of servicescapes is shaped by geographical proximity and physical accessibility but also by subjective feelings of belonging or alienation. In turn, these emotions are often underpinned by collective and historically informed representations of who belongs where (Dixon et al., 2022, 2025). In societies marked by entrenched ethno‐racial divisions, spatial representations often carry racial, ethnic or religious dimensions: some places are perceived as ‘belonging’ to particular groups, while others are viewed as more inclusive (Dixon et al., 2022; Huck et al., 2019). Perceptions of belonging are shaped by messages shared through media, political and other discourses, some of them motivated by a willingness to maintain racial or ethnic separation and hierarchies (Dixon & Durrheim, 2000), as well as group‐related visual and auditory cues such as flags, artwork, names and music (Dupont et al., 2024). Consequently, decisions about whether to engage in intergroup contact in urban spaces are a product of both practical and emotional factors. When individuals frequent places where they feel safe and at ease and avoid those they experience as alien or threatening, segregation patterns may be reproduced even in the absence of any legal enforcement.

Habitual contact patterns occur within space but also across time given the temporal endurance of place perceptions, both from a historical and life course perspective. Proshansky et al. (1983), who coined the concept of ‘place‐identity’, argued that aspects of the physical environment become incorporated into the self through accumulated experiences, memories and meanings associated with places that have supported individuals' social, cultural and emotional functioning. These experiences help explain why certain environments come to feel familiar, meaningful or comforting over time. Corroborating these insights, research has shown how the value of some places derives from their capacity to root users in family and cultural traditions (Dixon & Durrheim, 2004). The flip side of such positive place‐identity is that past experiences of negative intergroup contact, including incidents dating back several years and those involving close ingroup members rather than the self, can trigger feelings of place‐related threat and long‐term avoidance (Dixon et al., 2020; Dupont et al., 2024). Hence, individuals' experiences in urban spaces play an important role in determining whether or not they use them in the long run.

## The present research

Building on the growing body of research cited above, the present research sought to answer three research questions: (RQ1) How do youth intergroup interactions play out in everyday life?; (RQ2) How do urban spaces facilitate or inhibit youth intergroup interactions?; and (RQ3) What psychological processes underpin the relationship between space and intergroup contact? We situate our research in the context of Bradford, a site of integration focus in the UK Government's Integrated Communities Strategy Green Paper (HM Government, 2018). Bradford has the largest proportion of Pakistani origin individuals in England (20.3%; Office for National Statistics, ONS). In 2001, it became a flashpoint in riots linked to a number of causes including racism, antagonism toward the police, unemployment, poverty and far‐right mobilisation (Bagguley & Hussain, 2016). Twenty‐five years later, promoting social cohesion remains a local policy priority, and ‘getting along’ (promoting greater interaction) is a key pillar in Bradford City Council's (2018) Stronger Communities Together Strategy. We focus specifically on youth aged 16–18 who are negotiating intergroup relations at a critical period in which they are starting to make autonomous choices about which urban spaces to socialise in.

To understand young people's lived experiences, we adopt a qualitative participatory photography approach. This method is particularly suited to youth samples and theory development because it gives agency to participants to identify and represent the urban spaces that shape their social interactions. By anchoring later discussions in participant‐generated images, the method grounds reflection in subjectively meaningful contexts rather than researcher‐defined settings, eliciting spatially situated accounts of social interaction that might otherwise remain hidden. Participatory photography is especially well suited to examining how everyday environments intersect with psychological processes, as it enables us to capture the features of urban spaces that promote or prevent intergroup contact. To our knowledge, few studies have used this approach to investigate the relationship between everyday urban spaces and intergroup dynamics, and even fewer have done so with youth samples.

## METHOD

### Participants and recruitment

Two groups of Bradford youth were recruited through a number of grassroots organisations, youth groups and a college. Organization leaders invited 16–18‐year‐olds to join photovoice workshops and mixed‐ethnicity focus groups, plus optional walking interviews. The grassroots organisations included those that worked with women and girls, offering social, recreational and wellbeing activities, as well as those working on youth‐related sport initiatives. Youth groups included those organised by local council authorities. The college enrolled students from across Bradford's diverse neighbourhoods, meaning participants were likely to encounter peers from different backgrounds on a daily basis. This recruitment strategy was chosen to reach a diverse sample of youth from ethnic minority and majority backgrounds.

In total 33 youth aged 16–18 (13 Asian female, 3 Asian male, 9 White female, 2 White male, 3 White non‐binary, 1 Black female, 1 mixed Black/White female, 1 Arab female) took part in the photography workshops and focus groups with a subsample of 9 youth (2 Asian female, 2 Asian male, 1 White female, 1 White male, 1 White non‐binary, 1 Black female, 1 Arab female) opting to take part in walking interviews.

### Materials and procedure

Following ethical approval from the University of Bristol, data were collected using a photovoice approach (Sutton‐Brown, 2014). The first group (18 focus group and six walking interview participants) took part in May 2023; the second group (15 focus group and three walking interview participants) took part in June 2023. Young people joined photography workshops and focus group discussions at either a local community centre (Group 1) or a college (Group 2), followed by walking interviews.

#### Photography workshops

At the first meeting, young people were introduced to the research and gave informed consent. The project was framed as an exploration of everyday social life in Bradford, and specifically how places can help or hinder interactions between different ethnic groups. They then attended two photography workshops, each lasting two hours, facilitated by Shy B Photography, a Bradford‐based professional photographer with experience in youth community projects. This was followed by a 1‐week, participant‐led photography task where youth were asked to take photos of the places that shaped their social life beyond the home, school and work. In a third two‐hour workshop, participants selected and enhanced the four or five photos that they felt best reflected their interactions with people of different racial, ethnic or religious backgrounds. The photos were then printed out by the second author and used as prompts in one‐hour focus group discussions as well as for later photo exhibition in the local community to showcase youth perspectives.

Throughout the workshops and photomission, youth used a pre‐configured mobile phone including a camera and a bespoke photo editing app. Before use, youth were briefed on conditions of the phone and app use, such as avoiding taking photos of identifiable individuals or in places and at times where they did not feel safe. At the end of the third workshop, mobile phones were returned to the researcher, who uploaded the photos to a secure server, printed them out and brought them to the focus groups to be used as discussion prompts.

#### Focus group discussions and walking interviews

Focus groups were held with four ethnically mixed groups comprising three to six participants each, held in the same location as the workshops. Focus groups were unstructured such that there was no fixed interview schedule or set questions used. Rather, youth were encouraged to discuss the meaning of their own photos and comment on the places photographed by others. Conversations were then loosely steered by the researcher in line with our project aims to explore how public spaces enabled or prevented positive or negative intergroup contact. After the focus groups, youth were given new consent and feedback forms to indicate interest in taking part in a one‐hour individual walking interview with the same researcher. These interviews were also largely unstructured and conducted with a view to clarifying and specifying the place‐related perceptions discussed during the focus groups. Interviews took place within 4 days, following routes chosen by participants that included photographed places and some additional ones. Most remained within or near the city centre, but two participants chose large parks in the residential suburbs.

Participation was compensated with the mobile phone used during the study or with a £100 voucher, at young people's discretion. Youth who consented to a walking interview received an additional £25 voucher.

### Reflexivity

We adopted reflexive thematic analysis (RTA; Braun & Clarke, 2022) and consistent with this approach, we foreground reflexivity by acknowledging that our positionality as researchers shaped every stage of the research process. The project was designed, funding obtained and the final stages of the qualitative analysis and the subsequent manuscript write‐up conducted by the last listed author who is from Northern Ireland. Her experiences growing up in a socially divided context allowed for a nuanced understanding of youth experiences when it comes to navigating difference in a segregated city. At the same time, she was unfamiliar with the Bradford context and as such, held an outsider perspective. The focus groups and walking interviews, as well as additional analysis and parts of the writing, were conducted by the second listed author, a White immigrant to Britain, unfamiliar with Bradford. His outsider status may have encouraged openness in individual interviews but also may have contributed to a positivity bias in portrayals of intergroup experiences in Bradford. Initial data analysis, revision of themes through to later stages of the analysis and write‐up of parts of the manuscript was carried out by the first listed author, a British Asian from a diverse metropolitan area, who brought an insider perspective on navigating intergroup dynamics involving White British and British Asian communities. However, differences in age and regional background positioned him as an outsider in other respects, reflecting the nuanced and fluid character of insider/outsider status.

Rather than seeking a neutral perspective, we aimed for transparency and reflexivity in exploring the contextual determinants of youth intergroup contact.

### Analytic approach

The analysis was guided by our three research questions: (1) how do youth intergroup interactions manifest in everyday urban spaces? (2) how do urban spaces facilitate or inhibit these interactions? and (3) what psychological processes are involved? Data from focus groups and walking interviews were analysed together, since they largely offered complementary perspectives on the same places and interaction experiences. The photographs were not used as stand‐alone data sources but as interpretative aids when analysing focus group and walking interview data.

Building on RTA principles (Braun & Clarke, 2022), we treated analysis as an active, interpretative process shaped by researcher subjectivity and incorporated reflexive engagement with the team's diverse positionalities throughout (Berger, 2015). The process was iterative and recursive, going between familiarisation, coding, theme development and refinement, and involved repeated movement between the data, codes, candidate themes and theoretical arguments.

The focus groups and walking interviews were transcribed verbatim. The listed first author undertook immersive familiarisation with all transcripts and conducted inductive coding in NVivo. We then held team discussions to interrogate assumptions and identify theoretically relevant empirical patterns aligned with our research questions. Informed by these conversations, the listed first author returned to the dataset to re‐code segments and cluster some codes into broader themes, followed by further team meetings to refine and rework these themes. Findings were iteratively co‐written and revised by the first, second and last listed authors with a view to developing contact theory while remaining firmly grounded in participants' narratives.

## FINDINGS

Three interrelated themes capture young people's accounts of the contextual and psychological factors shaping their intergroup interactions in urban spaces: (1) Habitual contact and biographical ruptures; (2) The negotiation of place‐based belonging; and (3) Temporality, memory and spatial foundations of contact.

### Theme 1: Habitual contact and biographical ruptures

We use habit to refer to repeated interactions cued by stable contexts, and biographical ruptures to refer to life transitions that disrupt these routines. Biographical ruptures make new interactions possible but not inevitable, since comfort and familiarity can pull young people back to prior ties. In our data, servicescapes produced stable patterns of co‐presence that sustained existing ties through low‐effort routines, while biographical transitions expanded potential contact. Conversion of these opportunities into intergroup friendship depended on comfort, existing network inertia, and the institutional expectations, mixed‐group activities and inclusive norms that lowered the effort and social risk of intergroup interaction.

#### Habitual contact: Same places, same friends

Young people described a range of ways in which they regularly spent time with ingroup and outgroup friends in servicescapes. These included sitting and walking around, playing in the park, eating, shopping, using photo booths, playing games in an arcade, going to the cinema, attending concerts and other public events and taking the bus. These everyday activities were often combined within a single outing and described as long‐standing traditions or habits:

Me and my friends, they're like me, we all stick to one place. After I go to the cinema next week, if we feel hungry we could go to Nando's, it's right near the Cineworld, or we can just go to KFC. A few of us take the same bus home, so we'd be able to go home together so we won't be separated, we'll just be together for longer. (Group 1, walking interview 3, Asian, female)

This pattern shows how fixed routes and clustered venues reduced coordination effort and made it easy to keep the same group together across multiple activities. Youth in our sample mostly socialised in the city centre during lunch breaks or right after classes, with Broadway shopping centre and City Park (a large open area with water fountains in the middle, flanked by a monumental town hall and some restaurants) emerging as favourites. It was clear throughout the discussions held with young people that these servicescapes operated as routine meeting points, structuring who was likely to encounter whom and under what conditions. Features of these settings, such as co‐located low‐cost venues, seating and shelter, made it easy to hang out and to combine activities within a single outing. As such, servicescapes can support the maintenance of existing friendships, including ethnically diverse friendship groups, by enabling repeated use of the same locations with the same people, thereby reinforcing established social ties. At the same time, however, these routines may also limit exposure to unfamiliar others.

#### Biographical ruptures and contact opportunities

For many young people who had attended secondary schools in more segregated areas of the city, the transition to college had increased the leisure time they spent with diverse friends in the ethnically mixed urban spaces surrounding the college. We treat the move from school to college as a critical institutional rupture because it reorganised daily timetables, travel routes and proximate servicescapes. As one participant explained:

I come here more now but basically before […] in secondary school I would say my school was more South Asian so my friend group was more South Asian so we used to come here. But now I have gone to college and there are more people from different areas, different ethnicities, so I think I have expanded my social group. It is not like before that I would have excluded them but it is just because there were mostly Asians in secondary school. (Group 2, focus group 3, Asian, female)

This young person frames change as exposure rather than altered preference. The rupture created new co‐presence and expanded the feasible set of companions. Specifically, this account illustrates how early patterns of ingroup interactions were not necessarily driven by exclusionary intent, but by limited exposure. The transition created opportunities for co‐presence with new peers. Where these opportunities became repeated interaction outside class, they stabilised as intergroup friendships. The shift to a more diverse educational setting created new opportunities for contact, suggesting that transitions to more inclusive environments can help disrupt segregated friendship networks formed earlier in life. Where the college reinforced intergroup cooperation and set clear norms for inclusion, these ties spilled over into nearby unstructured spaces. Once outside the regulated setting, repeated leisure encounters helped the new intergroup bonds become self‐sustaining. Uptake appeared to vary with time and cost: longer after‐college periods in affordable venues seemed more likely to support intergroup mixing in comparison to brief, low‐spend lunch breaks.

#### Comfort, familiarity and the pull of prior ties

Not all young people, however, were equally receptive to these intergroup contact opportunities, and some acknowledged that the mostly ingroup networks they had developed in secondary schools had changed little since their enrolment in college:

I Would you go with people from certain backgrounds to some of those places more than others, would you say?

P I don't think so, no. Just my friend group, just from college, just for lunch.

I Yeah, and those friends, are they mainly from your background, from different backgrounds? What do you think?

P Mainly my background, to be fair, but I don't think that's a choice. It's just people I already knew, so I stayed friends with them in college.

I So you knew them from secondary school, or…

P Yeah, yeah. So it's more we've grown up together and then stayed as a friend group because it's easier than making new friends.

(Group 2, focus group 1, White, male)

Here, continuity reflects an effort‐saving strategy and affective ease. This effort economy helps explain why even critical ruptures did not reliably translate into new intergroup ties. This account reflects a broader pattern in which early friendship networks shaped by residential and school‐based segregation continued to structure habitual contact in a new environment. This suggests that while biographical ruptures can create new opportunities for contact, they do not guarantee change due to the practical ease and emotional need of maintaining existing ties.

In sum, intergroup friendship in this context is habit‐driven but opportunity‐constrained. Biographical ruptures, such as the move from school to college, can expand opportunities, but conversion into enduring intergroup ties depends on place affordances, institutional scaffolding and the effort economy of friendship formation. Interventions that alter routines, extend shared time windows and make mixed servicescapes easy to hang out in seem most likely to shift contact.

### Theme 2: The negotiation of place‐based belonging

Young people explained how deciding where to spend time in cross‐ethnicity friendship groups involved complex negotiations and deliberate efforts to understand their friends' feelings of belonging in specific urban spaces. This process often deepened their knowledge of which spaces were perceived by different groups as inclusive or exclusive:

My friend right, she's Black and she said that when she was in Lister Park she does feel like an outcast […] but if she went to Ladyhill Park she'd probably feel more comfortable. (Group 1, focus group 1, Asian, female)

Two factors were frequently mentioned as underpinning feelings of belonging, namely the activities unfolding in a place and perceptions of the outgroup's attitudes toward the ingroup in that place (meta‐perceptions).

#### Activity scripts and servicescape affordances

We treat activity scripts as tacit expectations about what is done in a place, by whom, and how, and servicescape affordances as features of venues (such as layout, food menu, alcohol policy, seating and location) that enable or constrain co‐presence, eating together and lingering. Where these cues signal inclusion of diverse ethnic groups, venues operate as everyday bridging spaces. Where they do not, ethnically mixed friendship groups are likely to go elsewhere. Activity scripts signal who a space is for through the activities it makes easy, while servicescape affordances shape how mixed‐ethnicity groups can share a place without sharing the same activity.

In most cases, group‐based preferences around the use of city centre spaces were linked to the activities they hosted. For example, some sports were seen as Asian (e.g., cricket) or White (e.g., rugby, skiing, skating, dangerous or expensive sports) (group 1, walking interview 2, Asian, male). Conversely, football was seen to be played by all:

Right next to the fountains there [are people] playing football every day […]. It is usually teenagers but there are quite often adults and they are very multicultural; so White people, Asian people and Black people all just playing football which is quite good. (Group 1, focus group 4, Black, female)

Activity scripts can thus mark spaces as appropriate for some groups more than others. Within servicescape affordances, commensality (eating together) was central to how youth assembled and sustained mixed groups. Because of its rich cultural meanings and centrality in youth's social life, food was a salient marker of group preferences around urban spaces. Youth sometimes described food outlets and options as exciting and attractive due to their ethno‐cultural associations. These positive responses could arise automatically when walking around the city and motivate youth to adjust their plans in order to explore a place offering ingroup food:

The other day me and my friends were walking around and we just saw this new Caribbean shop. There is no Caribbean food in Bradford that we have seen before and my friend got very excited because she is Jamaican. She hasn't seen anywhere Caribbean before. We went in though and it was quite nice. (Group 1, focus group 4, Black, female)

Youth also displayed willingness to accommodate their friends' tastes by visiting food‐based servicescapes they would not normally go to on their own:

If we go to a new restaurant or something we'll try each other's food and if we like it and if it's not spicy enough or it's too bland, you need more flavour, and we'll just have petty arguments about that. (Group 1, walking interview 3, Asian, female)

Young people's food preferences, notably their spice preferences, were shaped by their cultural heritage and upbringing. These differences became particularly apparent during shared meals, where navigating contrasting tastes often required thoughtful strategies for inclusion. One way in which youth managed group‐based differences in tastes was by having lunch in food courts offering a range of affordable options, and more generally by getting takeaway and then finding a place to sit together. However, some establishments were described as having wide cross‐group appeal – as bridging spaces able to connect across ethnic divides. Nando's, KFC and other chicken shops, as well as fish and chips, were seen as bringing together the broadest range of people. The latter in particular was regarded as having shed its associations with (an implicitly White) Englishness, at least in the context of Bradford:

Me and my friend group have a bit of a tradition. After we have an exam, we always go [to a fish and chip shop by City Park] for our lunch break […]. So we get the lunch break and we always go there and we have a very diverse class […]. And I wanted to show that there is a different group of people, even though it's your traditional fish and chips, which is your traditional English meal, there's a lot of different people who go there to get different stuff. And I think it's just very nice that it's almost not just English culture, but it's accessible by everybody as well. (Group 2, focus group 4, White, non‐binary)

Corroborating this account, another participant in the same focus group (British Pakistani and female) explained that she lived on a diverse street including White, Arab and Slovakian neighbours, as well as ‘all these other cultures’, and that her family had also developed a tradition of celebrating ‘fish and chip Friday’ with a White neighbour.

Food courts and to some extent the central City Square, surrounded by a number of eating venues, could be seen as inclusive or exclusive depending on the availability of halal food for Muslim youth or the presence or not of alcohol. Some spaces, such as a leisure centre combining an arcade, a bowling alley and a restaurant, nevertheless enabled sustained interactions across religious (and ethno‐racial) boundaries even if there was a presence of alcohol and the restaurant did not advertise halal food.

#### Meta‐perceptions and vigilance

Anticipated outgroup evaluation shaped whether contact felt feasible or effortful. Where young people expected scrutiny or microaggressions, interactions required vigilance and emotional regulation, which reduced the appeal of staying, returning, or deepening ties. Youth, particularly those identifying with an ethnic minority, also viewed some spaces as exclusive due to how they expected to be perceived and treated by outgroups in these spaces. The following account shows how Asian young people who wish to hang out with friends in pubs are sometimes made to feel unwelcome or uncomfortable by demeaning gestures (‘dirty looks’) and comments. While these do not necessarily amount to explicit exclusion or physical aggression, neither do they suggest any meaningful attempt to avoid appearing racist, which highlights their normative character in this particular space:

Wetherspoons is mainly for the White community and I've been there before with [a White friend] and I was getting dirties pulled at me like ‘why are you here?’, yeah, so it makes you feel uncomfortable but then again, I'm like well I've come here to celebrate something with my friend, I've not come for anybody else, so I'd rather just enjoy my meal with her. […] In terms of pubs they don't really say to you like ‘oh get out’ it's more like the sort of looks you get, and it makes you slightly uncomfortable. When I used to do swimming competitions, they used to have parties afterwards in pubs because everybody was White, me and my sister and my brother were like the only one of an ethnic background, we were the only brown people, and we did get hate crimes quite a bit. It wasn't like ‘oh get out you're not supposed to be here’ it was small comments that they'd make, like one guy said to my mum ‘oh did you come here by car or by donkey?’ and it didn't make any sense but he was trying to be racist and it was like well there's no need for it. (Group 2, walking interview 3, British Pakistani, female)

By obliging minority youth to remain vigilant, manage risk and regulate their emotions, these microaggressions undermine their capacity to enjoy otherwise convivial settings and may discourage them from staying or returning. Anticipated evaluation, rather than explicit exclusion, can therefore be sufficient to limit the depth and duration of cross‐group interaction.

In summary, place‐based belonging is negotiated through activity scripts and servicescape affordances, with food‐based and multi‐option venues acting as everyday bridging spaces when they accommodate group‐specific norms and tastes. These patterns show how contextual features of venues facilitate or inhibit intergroup interactions and how young people actively navigate these features to maintain cross‐group friendships.

### Theme 3: Temporality, memory and spatial foundations of contact

Young people's accounts suggest that long‐term memories shape present‐day intergroup contact through three processes: nostalgia, where positive memories attach to particular venues and make return visits feel meaningful; negative encounters, where past adverse incidents motivate future avoidance of a place; and habit maintenance, where childhood routines in how and where time is spent continue into adolescence. These processes help explain why some places become durable sites of mixing while others are steered clear of.

#### Nostalgia and durable place attachments

For many young people, the places they frequented in early childhood left vivid emotional imprints that influenced how they perceived and used urban spaces in adolescence. Several nostalgic memories shared by participants were rooted in experiences of servicescapes such as parks or leisure venues, frequented with relatives and close friends. The memories associated with them often reflected strong feelings of comfort, recognition and belonging. For some ethnic minority youth, it is the diversity of servicescape users that afforded safety and belonging. One Arab participant described how her family's early visits to a local park helped her feel at home after arriving in the UK:

R We came from Emirates. Do you know where Emirates is?

I Yes.

R I was born there. We came from there. Obviously my dad knew a bit of English from work but us, as siblings, no one knew English so parks were really the only place where we could be ourselves. Be our Arabic selves. […] Whenever family friends come over we just sit with them and then, because it's very – as I said Bradford community is safe and trustworthy. So, my mum would let all the kids go off into the actual park and you know, we'd all be safe and we'd all still have fun. […] No one could judge us. No one would have to speak to us. We could do whatever we want. Bring our own traditional food. Coffee whatever. It was just fun and it was yeah, fun for us.

[…]

I Did you come with the neighbours sometimes as well or mainly within your family?

R Firstly, within our family, because as I said, we didn't have no one. We didn't have family. Family only moved recently so we were alone so that's why park was always our first choice. But because it's a very diverse neighbourhood here. We found so many people from Arabic cultures. So many people from Pakistan. So, people Polish and Slovakian, Pakistani and even British. You find so many so what would happen is we would sit normally and we'd see another Arabic family from a distance and we'd just – that was like the first time I think Arabic kid with me on the swing and I went to my mum and I said there's an Arabic person here. I was like, mamma there's an Arabic person here. That was something big for us, because we thought Britain was you know, only English people. So even our closest family friends right now […], we met them here. We were sat in our usual spot and there was a family close by and I think they heard us talk Arabic, so they came over and they gave us their cake, that they made, and yeah, my mum and dad exchanged numbers and ever since then we've become friends.

(Group 2, walking interview 1, Arab, female)

This detailed, lively account suggests that early positive experiences in diverse settings can seed durable attachments to mixed venues, making later intergroup contact feel ordinary rather than exceptional. These emotionally relevant early experiences often laid the foundation for long‐term spatial routines, reinforcing attachment to places that felt familiar and safe. In some cases, this contributed to patterns of informal segregation, as young people continued to frequent spaces associated with their ingroup. However, this was not always the case, and both ethnic minority and White participants described childhood attachments to spaces that were diverse and inclusive. One young person reflected on growing up in a multicultural arcade:

I grew up playing in this certain arcade. It is quite diverse I would say. When you go in there are loads of kids from different races and religions and stuff but also adults with the kids. It is just quite nostalgic. (Group 1, focus group 4, White, female)

The fact that such spaces were remembered fondly and continued to be used into adolescence suggests that early positive experiences in diverse environments can foster durable attachment to diverse spaces. Here, nostalgia functions both as a reminiscence and as a practical guide, pointing young people back to settings where difference has been safely negotiated before.

#### Negative encounters and long‐term place avoidance

Not all memories were enabling of place use and intergroup contact. Where prior experiences involved hostility or shame, participants linked those incidents to the specific venues where they had occurred, and changed whether and how they visited those venues:

I don't like the people that you come across in the park at all. The people aren't friendly, let's say […] They try to approach you and talk to you and stuff. It makes you feel uncomfortable, but I go there with my little sister. She's four and I take her to the park because she enjoys it […] I'm just walking with my little sister [and] they just follow you and just start shouting and stuff. It's just really weird and you don't even know them, and they'll be young lads and you don't even know them. There's groups of them and then they'll just start saying really inappropriate stuff […] They always come out at 6pm, or, on the weekend, about 12 in the afternoon. So I avoid those times of going to the park because I know that there's weirdos there. (Group 1, focus group 2, Asian, female)

This account shows how remembered encounters can reconfigure place use, with young people learning to avoid particular places at particular times. For this participant, the combination of being young, female and from an ethnic minority raised the vigilance required, turning the park into a setting for being present but wary rather than relaxed and ready for mixing. Enduring place‐linked memories mean that even a single negative encounter can trigger avoidance that, if repeated, hardens activity‐space segregation (Dixon et al., 2020, 2022). Thus, encounter quality and meaning are important for shaping intergroup orientations.

#### Habit maintenance across life stages

Early spatial routines were often reproduced at later life stages, unless transitions introduced compelling alternatives and made new routines easy to maintain. Youth narratives illustrate how these habits can either reinforce patterns of segregation or foster sustained mixing over time. The following account illustrates a positive shift prompted by a routine primary school visit to a city museum:

We went to visit the Media Museum because in primary school we used to go every year because obviously it is a free thing, so me and my friends decided to go once after. Met a group of Black guys who were so nice and took pictures for us, so we hung around with them for the whole duration while we were there. We didn't know them, but they were so nice to us. (Group 1, focus group 3, White, non‐binary)

This example shows how a familiar and affordable setting can enable convivial intergroup contact with strangers, such as practical help with taking photos, creating an atmosphere of ease and supporting shared time on site. Hence childhood memories of place, particularly those with strong emotional or nostalgic significance, appeared to shape long‐term preferences for urban space use. While participants remained attached to some culturally homogenous spaces, they also developed (to different degrees) lasting connections to diverse settings that supported intergroup friendships. These findings underscore the cumulative and path‐dependent nature of everyday contact, highlighting how the timing and quality of early experiences can influence intergroup interactions well into adolescence.

## DISCUSSION

The present research aimed to explore how youth intergroup interactions play out in everyday urban spaces, how contexts facilitate or inhibit them, and what psychological processes are involved. To achieve this, we adopted a participatory photography approach including focus groups and walking interviews, in which both photographs and the locations visited served as prompts for eliciting rich, situated narratives. We find that group routines, place‐based belonging and childhood memories all influence contact engagement and experiences among young people growing up in an ethnically diverse city. Our study introduces a novel perspective by focusing on everyday intergroup contact that occurs within diverse friendship groups initially formed in educational settings rather than among strangers or acquaintances.

First, our findings illustrate how youth interaction experiences are shaped by habitual routines and how life transitions can rupture such routines. Youth indicated how they tended to go to the same places repeatedly and with the same people, which can both serve to maintain current friendships (whether diverse or not) as well as potentially restrict interactions across group boundaries and with unfamiliar others. Youth also highlighted how life transitions disrupted their established contact routines. Specifically, they noted how the move from school to college offered them new opportunities to make friends across ethnic divides and whilst some did not take up this opportunity, others did. This demonstrates the importance of contact opportunity and the conversion of opportunity into realised interaction for facilitating contact engagement in segregated societies where people are more likely to spend time with those who are similar to themselves (McKeown & Dixon, 2017). Intergroup contact in urban contexts, therefore, is both embedded in everyday spatial routines and sometimes disrupted by life transitions.

Although our data do not allow firm conclusions about systematic ethnic‐background differences, the narratives suggest that opportunities for contact engagement created by life transitions may be taken up differently across groups. The Asian participant in our study described expanding her network at college, whereas the White participant retained same‐ethnicity ties. Future research could see how systematic this difference is and explore the reasons, which may include social expectations around integration into White majority culture.

Second, our findings show how ethnically diverse friendship groups negotiate belonging in urban spaces and how inclusive spaces can facilitate and sustain friendship with outgroup peers. We found that youth spent a significant proportion of their time in servicescapes such as parks, shopping centres and restaurants, and that the use of these spaces was influenced not only by practical considerations such as proximity, affordability and time‐availability but also by how welcoming they were felt to be. Youth noted how ethnicity‐related cues in such spaces had implications for who spent time there and with which friendship groups. Proshansky et al. (1983) have highlighted the significance of ‘physical environment‐related cognitions’ in relation to place‐identity, and this seems to go hand in hand with ‘activity‐related cognitions’ in relation to what could be termed ‘activity‐identity’. Whilst related, perceptions of places and of the activities unfolding in them are also different. On the one hand, the specific services and products available in a space were seen as expressing which group(s) the space was primarily intended for. On the other hand, because a number of activities and products could often be enjoyed in the same place, young people could share a place without sharing an activity (such as by eating or avoiding certain types of food).

When deciding where to spend time with outgroup friends, therefore, young people were obliged to navigate and reconcile complex perceptions on which spaces are attractive and safe for different ethnic groups. From a theoretical perspective, what stands out is the importance of spatial belonging, safety and navigation in maintaining cross‐group friendships in urban spaces. From a practical perspective, findings suggest that positive intergroup interactions could be fostered by designing more affordable and ‘bridging’, multi‐activity spaces near educational settings, including food courts, public squares surrounded by a variety of restaurants, and multipurpose play areas.

Third, our findings demonstrate how intergroup contact in urban spaces was dependent upon past experiences and nostalgia. Youth explained how their time spent in urban spaces in the past had created significant memories that often determined whether and with whom they frequented those spaces. Whilst there is some overlap here with aspects of habit, we argue that the temporal nature of contact as pertaining to histories and nostalgia is set apart by the more deliberate choice of space use linked to meaningful, emotionally significant past experiences in that space. Evidence shows that people tend to gravitate toward places that evoke nostalgic feelings, with the act of reminiscing itself having a soothing, emotionally restorative effect (Oliver et al., 2024; Turner & Stathi, 2023). Importantly, past experiences, including those involving intergroup contact, are often remembered more positively than they were originally experienced, a phenomenon known as the ‘fading affect bias’ (Walker & Skowronski, 2009). Emotionally charged experiences, both positive and negative, were often recalled and connected by youth in our research to the place where they unfolded over a number of years, evoking persistent feelings of nostalgia or anxiety. In this sense, we find that both segregation and mixing are cumulative and path‐dependent processes, with earlier experiences having a greater impact on youth decisions around future interactions. This demonstrates the importance of understanding both the spatial and historical dimensions of everyday intergroup contact engagement to determine the conditions under which it is likely to occur.

## LIMITATIONS AND FUTURE DIRECTIONS

Despite the strengths of the present research in understanding the nature and conditions of everyday youth intergroup contact, a number of limitations should be acknowledged. The first is that our findings reflect the perspectives of a group that was predominantly Asian and female, which likely shaped the themes identified. Despite its richness, we are therefore unable to claim generalisable contextual or group‐based patterns from our data. Future research could explore how habitual, spatial, and temporal patterns of contact, shaped by experiences of belonging, vary across ethnic and gender groups.

Second, our data were collected over a relatively short period of time. As such, the study provides a snapshot of young people's experiences, during late spring, a period where outdoor experiences and places may be more salient. With emerging work exploring children's relationships through social network analysis (e.g. Burke et al., 2022), such an approach could be used in future research to evaluate the degree to which friendship networks are diverse and change over time across urban spaces. This would help clarify whether and how servicescapes hinder or facilitate cross‐ethnic friendships over a more sustained period and around the year.

Third, our research was limited by the age range of participants (16–18), meaning that we were unable to explore how interactions unfold across the lifespan and how different biographical ruptures influence contact engagement. Future research could use repeated cross‐sections or longitudinal data to examine how young people across different age ranges use social spaces and the factors that influence their positive and negative intergroup interactions in the city.

Fourth, whilst our participants reported on their interactions in the city, they tended to focus primarily on positive accounts which means we may have missed out on some negative forms of contact. There seemed to be some reluctance among young people to discuss negative intergroup experiences, which may be due to researcher positionality but also to implicit norms conveyed during the mixed photography workshops and focus groups (which preceded the individual walking interviews). Future research might consider using same‐ethnicity focus groups to encourage freer discussion. This should be done while carefully weighing the risk of inadvertently reinforcing ethnic divisions or antagonisms, which was a key reason we opted for mixed groups in this study.

## CONCLUSION

Our research illuminates the habitual, spatial and temporal aspects of intergroup contact among friends in late adolescence. We show how contact is experienced ‘on the ground’, its routinised nature and how servicescapes can alternatively act as facilitators and inhibitors of intergroup contact among youth growing up in an ethnically divided city. We demonstrate how urban spaces become sites of belonging or alienation, and how young people engage in complex negotiations around the use of space when hanging out in ethnically diverse friendship groups. Our research underscores the theoretical and practical need to explore the spatial and temporal nature of intergroup contact in unstructured urban spaces in order to determine how they can contribute to the maintenance of diverse friendship groups formed in more structured educational settings.

## AUTHOR CONTRIBUTIONS

**Sumedh Rao:** Conceptualization; writing – original draft; formal analysis; investigation; methodology. **Pier‐Luc Dupont:** Conceptualization; investigation; writing – original draft; methodology; formal analysis. **David Manley:** Conceptualization; funding acquisition; writing – review and editing. **Laura K. Taylor:** Conceptualization; funding acquisition; writing – review and editing; methodology. **Shelley McKeown:** Conceptualization; funding acquisition; writing – original draft; writing – review and editing; project administration; supervision; investigation; methodology.

## CONFLICT OF INTEREST STATEMENT

Last and corresponding author McKeown is Co‐Editor‐in‐Chief of the *British Journal of Social Psychology*.

## Data Availability

The data that support the findings of this study are available from the corresponding author upon reasonable request.
